# Metformin inhibits SUV39H1-mediated migration of prostate cancer cells

**DOI:** 10.1038/oncsis.2017.28

**Published:** 2017-05-01

**Authors:** T Yu, C Wang, J Yang, Y Guo, Y Wu, X Li

**Affiliations:** 1 Institute of Gene Engineered Animal Models for Human Diseases, Dalian Medical University, Dalian, China; 2 Institute of Integrative Medicine, Dalian Medical University, Dalian, China; 3 Department of Basic Science and Craniofacial Biology, New York University College of Dentistry (NYUCD), New York, NY, USA; 4 Biochemistry Section, Surgical Neurology Branch, National Institute of Neurological Disorders and Stroke, National Institutes of Health, Bethesda, MD, USA; 5 The Advanced Institute for Medical Sciences, Dalian Medical University, Dalian, China; 6 Department of Urology, New York University Langone Medical Center, New York, NY, USA; 7 Perlmutter Cancer Institute, New York University, Langone Medical Center, New York, NY, USA

## Abstract

Prostate cancer (PCa) is a leading cause of cancer-related death among men, largely due to incurable distant metastases. Metformin, the most common used anti-type-2 diabetes medicine, has been linked to reduced cancer risk and better diagnosis. We found that metformin was able to inhibit PCa cell migration, which correlates with tumor metastatic capability. The pathogenesis and progression of tumors are closely related to dysregulated gene expression in tumor cells through epigenetic alterations such as DNA methylation and histone modifications. We found that the level of SUV39H1, a histone methyltransferase of H3 Lys9, was reduced in metformin-treated PCa cells in a time-dependent manner. SUV39H1 overexpression increased PCa migration, whereas SUV39H1 depletion suppressed PCa cell migration. There is a positive correlation between SUV39H1 expression and PCa pathological stages. We further showed that both metformin treatment and SUV39H1 knockout in PCa cells can reduce integrin αV and β1 proteins, as well as their downstream phosphorylated focal adhesion kinase (FAK) levels, which is essential for functional adhesion signaling and tumor cell migration. Taken together, metformin reduced SUV39H1 to inhibit migration of PCa cells via disturbing the integrin-FAK signaling. Our study suggests SUV39H1 as a novel target to inhibit PCa cell migration.

## Introduction

Prostate cancer (PCa) is the most common malignancy and the second leading cause of cancer death in American men.^1^ It is estimated that there will be about 26 120 deaths from PCa in United States in 2016.^1^ The 5-years relative survival rate of PCa is almost 100% for local and region stages, but sharply drops to 28% for distant stage PCa. Currently, when PCa has spread to bone or other organs, it is incurable and causes significant mortality.^1, 2^ It remains critical to find effective approach on inhibiting PCa cell migration and invasion to prevent its spreading to other organs.

Metformin (1,1-dimethylbiguanide) as the first-line oral medication for the treatment of type-2 diabetes is cheap and wildly used.^3^ Multiple epidemiological studies and accumulated evidences have shown that metformin may reduce cancer risk and improve cancer prognosis and survival,^4, 5, 6, 7^ including PCa.^8, 9, 10^ Our previous work indicated that metformin inhibits salivary adenocarcinoma grow *in vitro* through cell cycle arrest and apoptosis,^11^ and targets c-MYC oncogene to prevent PCa growth *in vitro* and *in vivo*.^12^ In addition, metformin significantly inhibited PCa cell migration^13^ that is a critical step to cancer metastasis, which supports that metformin may inhibit PCa metastasis and progression in addition to its regulation on cancer cell proliferation and apoptosis. It has been reported that metformin may inhibit PCa growth via repressing mammalian target of rapamycin^14^ and androgen receptor signaling pathways.^15^ However, much remains to be understood about metformin’s anticancer properties and the direct molecular mechanism by which metformin inhibits PCa cell migration remains unclear.

It is notable that although the antitumor efficacy of metformin is supported by numerous epidemiological and pre-clinical evidence, metformin does not show effectiveness on PCa progression clinical trials. One possible mechanism may explain such discrepancy could be the dosage of metformin could not reach the minimal effective concentration to inhibit PCa cells in patients. Alternatively, finding the mediator of metformin’s inhibitory effect and direct targeting these molecule would be valuable for PCa treatment.

Epigenetic alterations, such as DNA methylation and histone modifications, including methylation, acetylation, phosphorylation and ubiquitination can directly affect gene expression, leading to activation or inactivation of signaling pathways to alter cell proliferation, apoptosis and/or motility in both benign and malignant cells.^16, 17, 18, 19^ Histone lysine methylation and DNA methylation are highly interrelated and interact on each other.^20, 21^ SUV39H1 is a histone methyltransferase that mediates H3K9 methylation, loss-of-function of which disrupts heterochromatin formation and may induce chromosome instability.^22, 23, 24^ The role of SUV39H1 in PCa cell mobility has not been studied and for the first time, we found that metformin’s inhibition of PCa cell mobility is associated with downregulation of SUV39H1. This observation indicates that SUV39H1 may mediate the effects of metformin on inhibiting PCa cell migration.

In this study, we revealed SUV39H1 as a target gene downregulated by metformin and investigated the role of SUV39H1 in PCa cell motility. Using both gain-of-function by overexpression, loss-of-function and rescue systems with CRISPR-Cas9 mediated knockout (KO) and re-expression of SUV39H1 in human prostate cell lines, our results indicate a novel role of SUV39H1 in regulating PCa cell migration.

## Results

### Metformin inhibits the PCa cell migration and SUV39H1 expression

**Figure 1:**
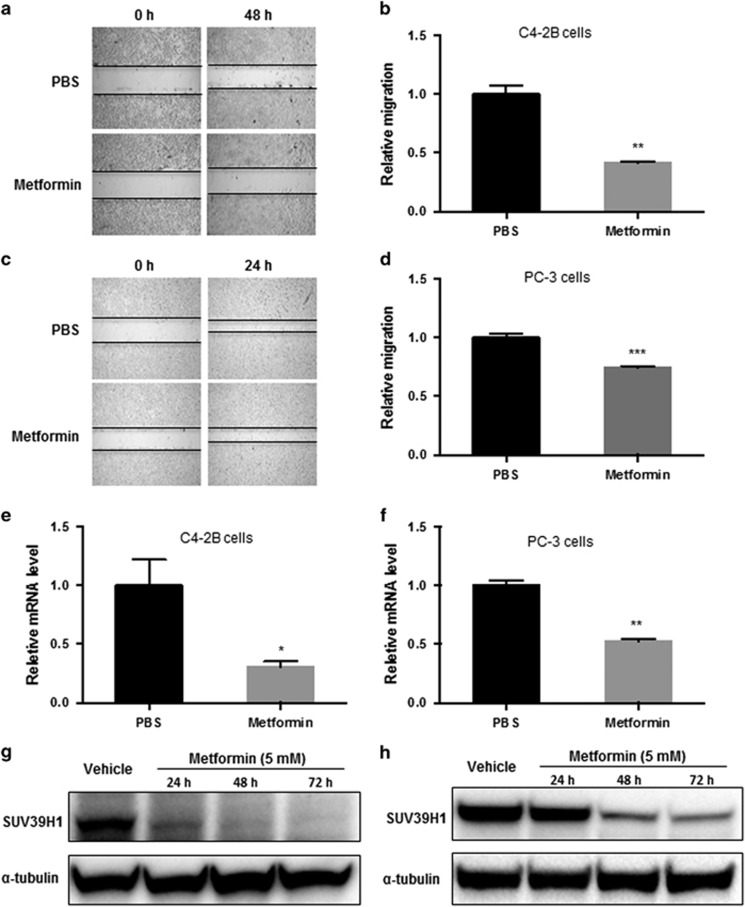
Metformin decreases the migration of PCa cells associated with the expression of SUV39H1. Wound-healing assays were performed to evaluate the migration of PCa cells (**a**, **b**, C4-2B cells; **c**, **d**, PC-3 cells) treated with 5 mM of metformin or PBS. Relative migration was calculated with migration distance measured under microscope. The quantitative PCR analysis of relative SUV39H1 mRNA level in C4-2B (**e**) and PC-3 (**f**) cells treated with metformin (5 mM) or PBS for 48 h. C4-2B (**g**) and PC-3 (**h**) cells were treated with metformin (5 mM) for 24, 48 and 72 h or PBS (vehicle). Whole-cell lysates were used for western blot assays. And α-tubulin was used as a loading control. Data shown are mean±s.e.m. (*n*⩾3). *P<0.05; **P<0.005; ***P<0.0005.

To assess if metformin inhibits the migration of PCa cells, we treated two metastatic PCa cell lines, an osteoblastic (C4-2B) and an osteolytic (PC-3) metastatic cell line, with metformin for 48 h and measured the cell migration. As shown in Figure 1, metformin inhibited cell migration in both PCa metastatic cell lines according to the wound-healing assay (Figures 1a–d). Metformin decreased the relative migration rate by 59% in C4-2B cells and 26% in PC-3 cells. Meanwhile, metformin significantly reduced the mRNA level of SUV39H1, a histone methyltransferase of histone 3 lysine 9 (H3K9) by 69% in C4-2B (Figure 1e) and 47% in PC-3 (Figure 1f) PCa cells determined by semiquantitative PCR assays. Using actinomycin D, which inhibits transcription through its binding to DNA at the transcription initiation complex and preventing elongation of RNA chain,^25^ we found metformin did not reduce the stability of SUV39H1 mRNA (Supplementary Figures S1a and b). Therefore, the reduction of SUV39H1 mRNA by metformin should be at transcription level. Further, we applied Cycloheximide (CHX) assess whether metformin regulates SUV39H1 directly or indirectly. CHX suppresses the *de novo* protein synthesis in eukaryotes by inhibiting the binding and releasing of transfer RNA from ribosome. If metformin reduction of SUV39H1 mRNA is indirectly mediated by other proteins, after the removal of metformin, CHX is likely to delay the restoration of SUV39H1 mRNA levels. We used linear regression to analyze the differences between the slopes representing the RNA recovery speed in cells pre-treated with phosphate-buffered saline (PBS) or metformin. In PC-3, the differences between the slopes was moderate (Supplementary Figure S1c), whereas in C4-2B cells the difference was significant (Supplementary Figure S1d). This result indicates that the reduction of SUV39H1 mRNA levels by metformin is likely to be indirect and relies on *de novo* protein synthesis at least in C4-2b cells. Metformin inhibition of SUV39H1 expression in a time-dependent manner in both cell lines with a rapid and marked reduction in C4-2B cells after 24 h treatment (Figure 1g) and a significant reduction in PC-3 cells after 48-h treatment (Figure 1h).

### SUV39H1 overexpression promoted C4-2B cell migration

**Figure 2:**
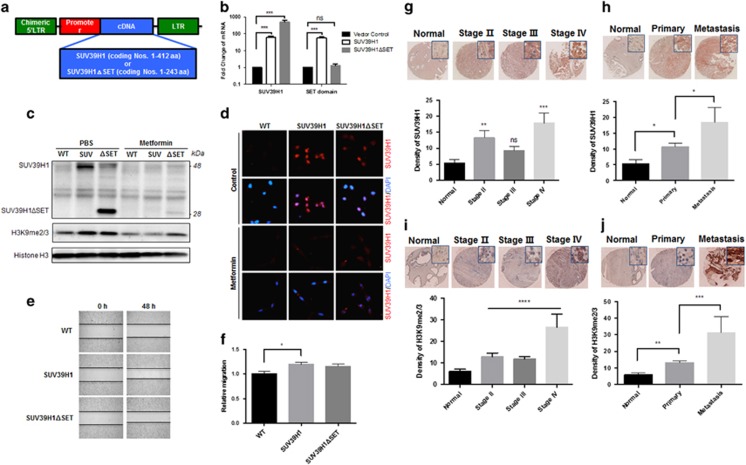
SUV39H1 overexpression promotes migration of C4-2B cells and PCa progression. (**a**) Construction of vector with SUV39H1/SUV39H1ΔSET. (**b**) Real-time quantitative PCR of the expression of SUV39H1 and SET domain in C4-2B control, SUV39H1 and SUV39H1ΔSET overexpression cells. (**c**) Protein levels of SUV39H1 and SUV39H1ΔSET in C4-2B WT, SUV39H1 (SUV) and SUV39H1ΔSET (ΔSET) overexpressing cells treated with PBS or metformin (5 mM). Wound-healing assay showed cell migration of C4-2B WT, SUV39H1 and SUV39H1ΔSET overexpressing cells (**e**, **f**) after 48 h and relative migration was calculated after quantified. Data shown are mean±s.e.m. (*n*=3). *P<0.05. (**d**) Immunofluorescence staining of C4-2B cells expressing SUV39H1 and SUV39H1ΔSET using anti-SUV39H1 (red) antibody, as well as DAPI (blue). Representative immunohistochemistry (IHC) images of PCa tissue (stage Δ, *n*=14; stage Δ, *n*=52 and stage Δ, *n*=14), normal prostate tissue (normal, *n*=14), primary PCa tissues (primary, *n*=72) and metastatic adenocarcinoma from prostate (metastasis, *n*=8), and AEC mean optical density as the measure of expression of SUV39H1 (**g**, **h**) or H3K9me2/3 (**i**, **j**). Data shown are mean±s.e.m. *P<0.05; **P<0.005; ***P<0.0005; ***P<0.0001; NS, not significant with *post hoc* *t*-test after analysis of variance (ANOVA).

SUV39H1 was known as part of an epigenetic machinery via its methyltransferase activity. However, whether and how SUV39H1 mediates metformin’s inhibitory effect on PCa cell migration has not yet been investigated. First, we decided to investigate whether SUV39H1 is able to regulate the cellular mobility in PCa cells. Using C4-2B cells, we generated two PCa cell lines with stable overexpression of different size SUV39H1, one is the full-length SUV39H1 (SUV) comprising 412 aa and another one is a C-terminally truncated SUV39H1 comprising 243 aa without the SET domain (ΔSET) (Figures 2a–c). We confirmed that the SUV39H1 and SUV39H1ΔSET are highly expressed in the corresponding stable cell lines. Interestingly, metformin treatment reduced the protein levels of both the full-length and the truncated SUV39H1 to the similar level of control cells according to western blotting (Figure 2c) and immunofluorescence staining (Figure 2d). The overexpression of SUV39H1 did not affect the PCa cell proliferation (Supplementary Figure S2), but significantly increased the migration of C4-2B cells. Of note, SUV39H1ΔSET overexpression only showed a moderate increase in cell migration but not significant compare with SUV39H1 full-length overexpression cells (Figures 2e and f). It seemed that the capability of SUV39H1 in promoting cell migration was correlated with their methyltransferase activity indicated by H3K9me2/3 levels (Figure 2c) as such stimulation is blunted in SUV39H1ΔSET overexpressed cells lacking methyltransferase activity.

Importantly, the clinical relevance of the aberrant SUV39H1 expression in PCa development was supported by immunohistochemistry staining of SUV39H1 and H3K9me2/3 in PCa tissue microarrays (Figures 2g–j). SUV39H1 (Figure 2g), as well as H3K9me2/3 (Figure 2i) levels were positively correlated with the progression of PCa stages in patients. Notably, the levels of SUV39H1 (Figure 2h) and H3K9me2/3 (Figure 2j) were both elevated significantly in metastatic PCa samples. These novel observation echoes the regulatory effect of SUV39H1 in PCa cell migration through H3K9 methylation and provides the direct and strong evidence supporting the role of SUV39H1 in the regulation of PCa progression and metastasis.

### SUV39H1 directly regulates PC-3 cell migration

**Figure 3:**
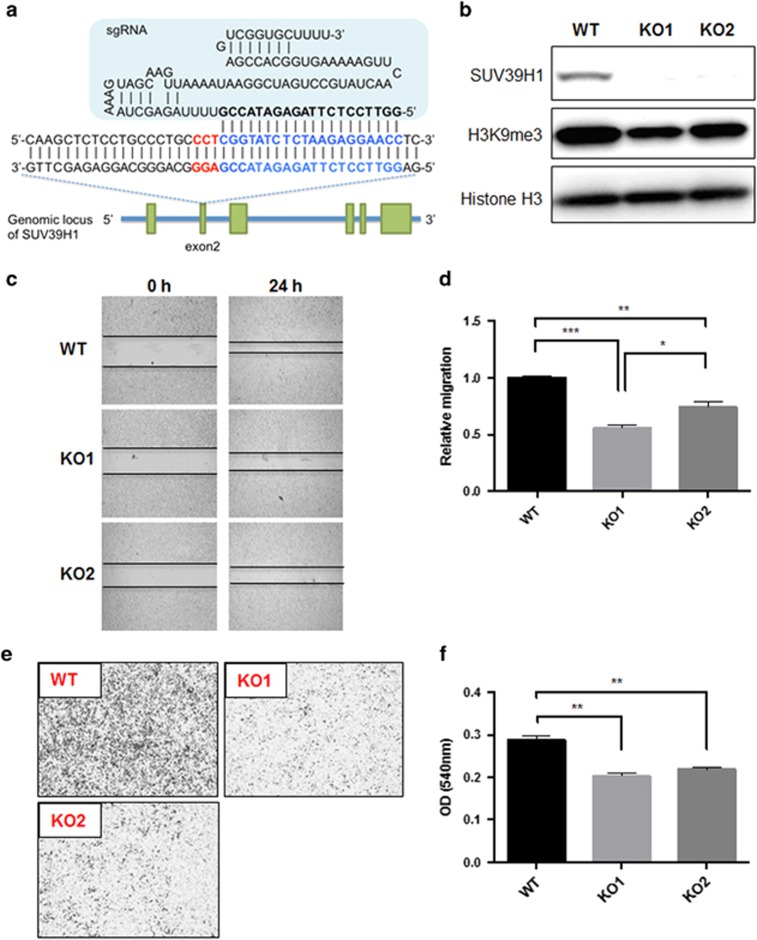
SUV39H1 deficiency decreases cell migration of PC-3 cells. (**a**) Schematic diagram of sgRNA binding to the target sites within SUV39H1 genomic sequences. Target site is highlighted in blue and bold with the PAM motif in red. (**b**) Confirmation of two SUV39H1-KO cell lines (KO1, KO2) by western blot. Cell migration was measured with wound healing assay (**c**, **d**) and transwell assay (**e**) and quantified with crystal violet staining (**f**) that shows optical density (OD) values served as relative migration. Data shown are mean±s.e.m. (*n*=3). *P<0.05; **P<0.01; ***P<0.0005.

To further determine whether SUV39H1 methyltransferase activity is essential to its ability in promoting cell migration, we further generated SUV39H1 KO cells and test whether re-expression of SUV39H1 or SUV39H1ΔSET can rescue/enhance cell migration. Here, we took a novel gene editing approach using CRISPR-Cas9 system to KO SUV39H1 gene in PC-3 cells and obtained two independent SUV39H1 KO cell lines (KO1 and KO2) (Figures 3a and b and Supplementary Figure S3). As expected, both KO1 and KO2 cells demonstrated declined H3K9me3 level because of the deletion of SUV39H1 expression. Both SUV39H1-KO cell lines showed significantly reduction in cell migration based on the wound-healing (Figures 3c and d) and transwell assays (Figures 3e and f). Meanwhile, cell proliferation is not affected in the KO clones (Supplementary Figure S4a), which is consistent with our previous observation in C4-2B cells (Supplementary Figure S2) that SUV39H1 is able to regulate PCa cell migration but not proliferation. In addition, the absence of SUV39H1 blunted the inhibitory effect of metformin (Supplementary Figure S4b), which further supports SUV39H1 mediating metformin’s inhibitory effect.

**Figure 4:**
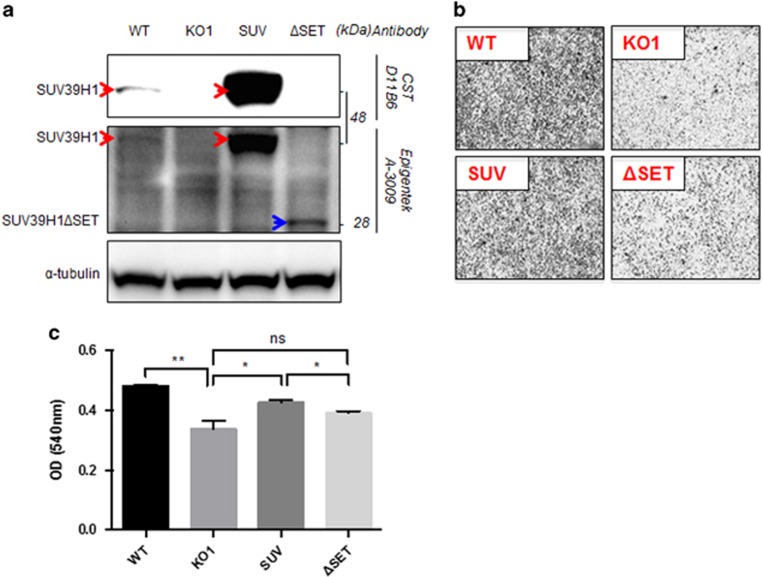
SUV39H1 re-expression rescues cell migration defect of KO cells. (**a**) Protein expression level of SUV39H1 and SUV39H1ΔSET in corresponding cell lines. (**b**, **c**) Cell migration of PC-3 WT, KO1, SUV39H1 (SUV)/SUV39H1ΔSET (ΔSET) re-expressing KO cells was shown with migrated cells dentistry in transwell assay quantified with crystal violet staining. Data shown are mean±s.e.m. (*n*=3). *P<0.05; **P<0.01.

When we re-expressed full-length SUV39H1 or SUV39H1ΔSET in the KO cells via stable infection of retrovirus (Figure 4a), the cell migration was rescued by re-expression of SUV39H1 but not by re-expression of SUV39H1ΔSET in the KO cells (Figures 4b and c). Therefore, SUV39H1’s methyltransferase activity is vital for its regulatory role in cell migration and the reduction of SUV39H1 expression or loss-of-function mutation impairs PCa cell migration.

### SUV39H1 regulates the integrin signaling

**Figure 5:**
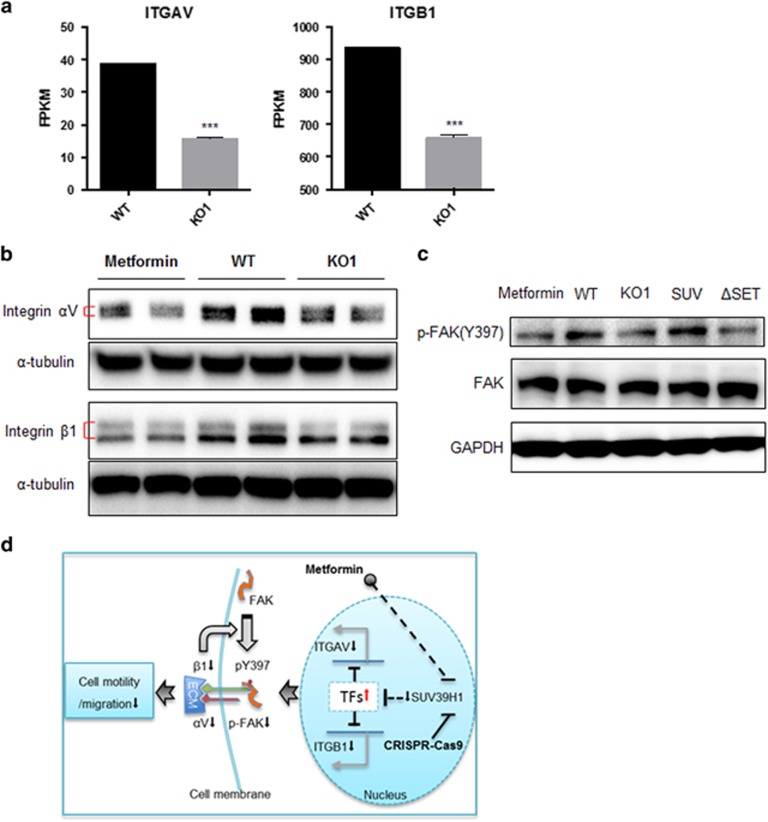
Both metformin and SUV39H1 deficiency suppress integrin-FAK signaling. (**a**) The mRNA levels of ITGAV and ITGB1 were reflected by FPKM (fragments per kilobase of exon per million fragments mapped) in RNA-seq data of PC-3 WT and KO1 cells. ***P<0.01. (**b**) Protein levels of integrin αV and β1 are reduced in SUV39H1-KO cells. (**c**) Immunoblot of p-FAK (Y397) and FAK in corresponding cell lines. (**d**) The work model shows metformin-induced SUV39H1 reduction decreases the motility of PCa cells via inhibiting integrin-FAK signaling.

To understand the downstream signaling pathways regulated by SUV39H1 expression in PCa cells, we compared the transcriptomes of the wild-type (WT) and SUV39H1 KO cells by RNA-sequencing analysis to predict the changes in signaling pathways by the deletion of SUV39H1. Interestingly, cell adhesion molecules and extracellular matrix (ECM)–receptor interaction pathways were among the top 10 biological pathways that changed significantly between WT and KO cells (Table 1). This result further supported the regulatory role of SUV39H1 in cell migration. Among the common genes represented in both pathways, we noticed that integrin αV (ITGAV) and integrin β1 (ITGB1) mRNA levels were reduced significantly in SUV39H1-KO cells (Figure 5a). ITGAV and ITGB1 are genes encoding proteins forming integrin heterodimers that bind ECMs as ligands and transduce extracellular signals into cytoplasm to induce focal adhesion kinase (FAK) auto-phosphorylation at Tyrosine (Y397). This progress has a vital role in cell shape, cell motility and migration.^26^ Indeed, we found that the reduction of SUV39H1, caused either by metformin administration or gene knocking-out, led to the reduction of integrin αV and β1 proteins (Figure 5b), as well as the downstream effector p-FAK (Y397) level (Figure 5c). As expected, the p-FAK level was able to be rescued by re-expression of SUV39H1 but not SUV39H1ΔSET in the KO cells. These data demonstrate the regulatory mechanism of SUV39H1 on cell migration through regulating integrin-FAK signaling in PCa cells.

SUV39H1 was reported as a suppressor through heterochromatin formation and heterochromatic gene silencing.^27, 28^ It is likely that the genes such as ITGAV and ITGB1 that are downregulated in SUV39H1-KO cells are indirectly regulated by SUV39H1. We screened the common transcription factors, which can bind to the promoter regions of ITGAV and ITGB1 among the 6544 SUV39H1-regulated genes in RNA sequence (RNA-seq) data (Supplementary Figure S5). We found 18 of them are capable to bind to both ITGAV and ITGB1 promoter regions. Further bioinformatics analysis revealed that there are six SUV39H1-suppressed transcription factors which have been reported as suppressors of their target genes’ expression (Supplementary Table S1). Therefore, the regulation of ITGAV and ITGB1 gene expression is likely be mediated through the silencing of the inhibitory transcription factors by SUV39H1.

Taken together, as depicted in Figure 5d, our results show a novel role of SUV39H1 in regulating cell migration. Importantly, it may mediate the inhibitory effect of metformin in PCa cell migration. Reduction of SUV39H1 could decrease the expression levels of ITGAV and ITGB1 through up-regulation of transcription suppressors, which eventually led to the decrease of integrin αV and β1 heterodimer formation. Indeed, the integrin signaling activity suppression was indicated by the reduction of p-FAK (Y397). Such disturbance of the integrin-FAK signaling contributes to the reduction of cancer cell motility and migration.

## Discussion

The inhibition of metformin in cancer cell proliferation and cancer growth has been reported previously by our group and others.^11, 12, 29, 30^ It remains unclear whether metformin directly inhibits cancer cell migration and cancer progression independent of its inhibition in cell proliferation. In this study, we prove that metformin inhibits different types of PCa cell migration and identified SUV39H1 as a target gene suppressed by metformin in PCa cells. We further revealed a novel function of SUV39H1 in regulating PCa cell migration via an integrin-FAK signaling. Using two metastatic PCa cell lines and several gene-editing techniques, our results made a direct connection between the expression of SUV39H1 and PCa cell migration ability. Further, metformin and SUV39H1 was demonstrated to regulate cell migration via a mechanism mediated by integrin αV, β1 and FAK auto-phosphorylation using RNA-seq and western blotting assays. To the best of our knowledge, this is the first report on the molecular mechanism by which SUV39H1 modulates PCa cell migration and metformin targets SUV39H1 to suppress PCa cell migration.

SUV39H1 is the first identified histone methyltransferase in human-mediating dimethylation and trimethylation of H3 at lysine 9 (H3K9me2/3),^22, 31, 32^ possessing a chromodomain and a SET domain originally identified in three *Drosophila* genes, Su(var)3-9, En(zeste) and Trithorax that are involved in epigenetic processes.^33^ The SET domain performs the catalytic activity, whereas the chromodomain has a role in histone H3K9me2/3 recognition.^34^ In our study, we found that cell migration was closely associated with the level of SUV39H1 but not SUV39H1ΔSET, which implies that SET domain-mediated H3K9 methylation has an important role in cell migration. Methylation was originally thought to silence gene expression. However, recent studies suggest that outcome of methylation on gene expression is much more complicated in general. It is not surprising to observe a profound impact of deleting SUV39H1 on gene expression in PCa cells. According to our RNA-seq data, there are 4142 genes upregulated and 2402 genes downregulated significantly (*P*<0.05) in KO1 cells compared with PC-3 WT cells. These data indicate that the H3K9 methylation by SUV39H1^35, 36, 37^) is able to directly suppress gene expression of some genes, but also able to stimulate gene expression. More studies are required to confirm whether SUV39H1 upregulates certain gene expression through inhibiting the upstream suppressors of these genes, like inhibitory transcription factors.

Integrins are a family of vital transmembrane cell surface adhesion receptors, comprising α and β subunits and having an important role in cell–cell adhesion and cell–ECM interactions,^26, 38, 39^ which were two of the most impacted biological pathways by SUV39H1 deletion (Table 1). The dysregulated expression of integrins have been linked to the aggressiveness in many tumors in which overexpression of integrins are associated with increased cell migration and tumor invasion.^40, 41, 42, 43, 44, 45, 46, 47^ The binding of ECM triggers integrin clustering and the formation of focal adhesions. Then, as one of the first downstream components to be activated by integrins,^48^ FAK becomes autophosphorylated at Y397, subsequently leading to activation of Src family kinases and other related signaling pathways, which regulates cell migration and invasion.^49, 50^ Cells with elevated levels of activated FAK exhibited increased migration, whereas cells with declined levels of activated FAK exhibited decreased migration.^51, 52^ Moreover, rather than kinase activity, Y397 phosphorylation of FAK is the key determinant of cell migration.^53^ The fact that p-FAK (Y397) level was closely correlated with the level of SUV39H1, strongly suggested the integrin-FAK signaling mediated metformin’s inhibition on PCa cell migration.

In conclusion, our data reveal a molecular mechanism by which SUV39H1 modulates PCa cell motility and demonstrate that metformin inhibited SUV39H1-mediated cell migration in PCa cells. This study supports targeting SUV39H1 as a novel strategy to reduce PCa cell migration and invasion.

## Materials and methods

### Reagents and cell culture

We purchased metformin from Calbiochem (Darmstadt, Germany), methanol and 10% neutral buffered formalin from Fisher Scientific (Kalamazoo, MI, USA), Dulbecco’s PBS and bovine serum albumin from Sigma-Aldrich (St Louis, MO, USA), RPMI-1640 medium and fetal bovine serum from Mediatech (Manassas, VA, USA), Actinomycin D from Life Technologies (Carlsbad, CA, USA) and CHX from Tocris Bioscience (Bristol, UK).

The antibodies against SUV39H1 (D11B6), GAPDH (D16H11), histone H3, di/tri-methyl-histone H3 (Lys9) (6F12), integrin αV and β1 (D2E5), anti-rabbit IgG (H+L), F(ab’)_2_ Fragment (Alexa Fluor 488 Conjugate) and anti-rabbit IgG (H+L), F(ab’)_2_ Fragment (Alexa Fluor 594 Conjugate) were all purchased from Cell Signaling Technology (CST) (Danvers, MA, USA). We used anti-SUV39H1 (A-3009) from Epigentek (Farmingdale, NY, USA) and α-tubulin (TU-02) antibody from Santa Cruz Biotechnology (Dallas, TX, USA), respectively.

We purchased human PCa cell line PC-3 from American Type Culture Collection (ATCC, Manassas, VA, USA) and received C4-2B cells as a gift from Dr Laurie McCauley (University of Michigan). Both cells were maintained in RPMI-1640 supplemented with 10% fetal bovine serum, 100 U/ml penicillin and 100 μg/ml streptomycin, and incubated in a 5% CO_2_ humidified incubator at 37 °C. We recently authenticated the origin of the cell lines. We conduct periodical checking to confirm they are mycoplasma-free using PlasmoTest—Mycoplasma Detection Kit (InvivoGen, San Diego, CA, USA).

### Gene KO with CRISPR-Cas9 system

SUV39H1 KO cell line was created by cloning SUV39H1 sgRNA (5′-GGTTCCTCTTAGAGATACCG-3′, targeting exon2) into George Church’s vector system (Addgene, Cambridge, MA, USA, plasmid#41824) followed by co-transfection with hCas9 (Addgene plasmid#41815) and pEGFP-C1 into PC-3 cells. Two days after transfection, green fluorescent protein-positive cells were sorted and plated into 96-well plates. Single colonies were expanded and screened for KO with the following primers: forward 5′-GATTTGGGGTCCCCTTTGA-3′ and reverse 5′-CCCTTTGGAAACAGATGTGGG-3′. The PCR product is 318 bp, and is cut by BsaJI into 151 and 167 bp. The homozygous clones were then confirmed by western blot.

### Generation of stable overexpressing cell lines and re-expressing cell lines

To make stable cell lines, SUV39H1-gi-EcoF (5′-gagaatcctggaccagaattcATGGCGGAAAATTTAAAAGG-3′) and SUV39H1-gi-XhoR (5′-gtgctggcggccgcctcgagCTAGAAGAGGTATTTGCGG-3′) or SUV39H1-gi-XhoR749 (5′-gtgctggcggccgcctcgagCTAGCGGAAGATGCAGAGGTC-3′) primers were used to PCR amplify the SUV39H1 or SUV39H1ΔSET and cloned into pBabe-puro at *Eco*RI/*Sal*I sites (*Sal*I and *Xho*I produce compatible overhang) by Gibson kit (NEB). The resulting retrovirus constructs were transfected into 293T cells together with helper plasmids to make virus. Virus was then used to infect corresponding cells with puromycin selection to make stable cell lines.

### Migration assays

We evaluated the migration and motility of PCa cells with two approaches: wound-healing and transwell assays. To conduct wound-healing experiment, we seeded cells in six-well plates. Upon the cells reached confluence, we used a sterile 1 ml pipette tip to scratch the monolayer and created a wound. After washing with PBS, we took photographs under a phase-contrast microscope (EVOS FL Cell Imaging System, Life Technologies). The cells were fed with fresh medium with 1% fetal bovine serum and incubated for 24 or 48 h before we took the photographs again. The relative cell migration distance was calculated by deducting *d*_24h/48h_ (the final distance) from *d*_0_ (the initial distance) and normalizing to (*d*_0_- *d*_24h/48h_) of the control groups.

We performed the Transwell assays with Transwell Permeable Supports (Corning, Tewksbury, MA, USA, #3421). First, we seeded the cells with 100 μl medium in the inside compartment of the upper Transwell inserts and fed the lower compartment with 600 μl medium supplemented with 1% fetal bovine serum. The cells were cultured for 48 h before they were fixed with 10% formalin and stained with 0.05% crystal violet (Invitrogen, Carlsbad, CA, USA). Then the upper cells and the remaining dye were carefully removed with cotton swabs. We took the photographs of the cells migrated to the lower compartment under a phase-contrast microscope. At the end, we dissolved the dye with methanol and measured the optical density at 540 nm to quantitate the amount of migrated cells.

### Cell proliferation

Cell proliferation was assessed with crystal violet staining. Briefly, 1.5 × 10^4^ cells (PC-3) or 2 × 10^4^ cells (C4-2B) cells per well were seeded in a 48-well plate. After 72-h incubation, the cells were washed with PBS, fixed with 10% formalin and stained with 0.05% crystal violet. Following twice with tap water washing to remove the extra dye and air-dry, we first added 100 μl methanol to solubilize the dye trapped in the cells, then transferred an aliquot from the 100 μl of each sample to a new 96-well plate and at last read the plate at OD540.

### Immunohistochemistry and tissue microarray analysis

The immunohistochemical staining was performed on the tissue microarrays (PR956) purchased from US Biomax (Rockville, MD, USA) using a HistoMouse-Plus Kit (R&D Systems, Inc., Minneapolis, MN, USA). Aminoethyl carbazole chromogen was used for signaling detection. Both primary antibodies against SUV39H1 and di/tri-methyl-histone H3 (Lys9) (H3K9me2/3) were used. The tissue microarray slides were counterstained with hematoxylin and scanned using Leica SCN400F (Leica Microsystems, Wetzlar, Germany) in the Histopathology Core at New York University (National Institutes of Health, Bethesda, MD, USA). Medical Center. We used ImageJ 1.48v (National Institutes of Health, Bethesda, MD, USA) to assess the mean aminoethyl carbazole-positive optical density of the entire area as the measure of SUV39H1 or H3K9me2/3 expression levels in each sample.^54^

### RNA stability and regulation of transcription

Actinomycin D (10 μg/ml) was administrated to PCa cells with metformin (5 mM) or PBS treatment for 24 h. The SUV39H1 mRNA levels were evaluated at the indicated time points and normalized to the mRNA level of each treatment group without the presence of actinomycin D. Using linear regression, we analyzed the differences between the slopes representing RNA degradation speed in cells with PBS versus metformin treatment.

CHX (50 μg/ml) was administrated to cells pre-treated with PBS or metformin (5 mM). Total RNA was extracted at 0, 1, 3 h post CHX addition and SUV39H1 mRNA levels were measured by real-time quantitative PCR and normalized to that from cells without CHX treatment. Using linear regression, the differences between the slopes of treatment groups were analyzed to reflect the recovery of transcription rate the removal of metformin to assess the impact of CHX on the restoration of SUV39H1 mRNA levels.

### Western blotting assay and immunofluorescence

We extracted the proteins from total cell lysates using RIPA lysis buffer (Thermo Scientific, Rockford, IL, USA), and nuclear proteins with EpiQuik Nuclear Extraction Kit (Epigentek, Farmingdale, NY, USA). The protein concentrations were determined with a BCA protein assay kit (Thermo Scientific). We used Bolt LDS sample buffer (Thermo Scientific) or sodium dodecyl sulfate sample buffer (2% sodium dodecyl sulfate, 62.5 mM Tris-base (pH 6.8), 10% glycerol, 5% β-mercaptoethanol and 0.005% bromophenol blue) to denature the proteins before sample loading for gel electrophoresis and membrane transferring as described previously.^55, 56^ The signals were detected with ECL Western Blotting substrate (Thermo Scientific) with a ChemiDox XRS system (Bio-Rad Laboratories, Inc., Hercules, CA, USA).

As described previously,^55, 56^ for immunofluorescence, we seeded 2 × 10^4^ cells per well into 24-well plates. After 48-h culture, the cells were washed and processed to overnight primary antibody incubation at 4 °C. The fluorochrome-conjugated secondary antibody was incubated the next day in dark for 1 h at room temperature. DAPI staining was conducted to visualize the nuclei.

### RNA extraction, quantitative real-time PCR and RNA-seq

Total RNA from PC-3 WT and SUV39H1-KO cells was isolated and purified using RNeasy Plus Mini Kit (Qiagen Sciences, Germantown, MD, USA, #74136) and the complementary DNA samples were obtained afterward by reverse transcription with a Taqman reverse transcription kit (Applied Biosystems, Carlsbad, CA, USA). We performed semiquantitative PCR using the SYBR green super mix (Applied Biosystems) on a CFX384 Touch qPCR System (Bio-Rad Laboratories, Inc.). Primers were as follows: SUV39H1 sense 5′-CCGCCTACTATGGCAACATCTC-3′ SUV39H1 antisense 5′-CTTGTGGCAAAGAAAGCGATGCG-3′.

RNA-seq was performed in the Genome Technology Center of New York University Medical Center, on an Illumina HiSeq2500 sequencer (Illumina Inc., San Diego, CA, USA). RNA-seq data were analyzed with the Illumina BaseSpace apps *TopHat Alignment* and *Cufflinks Assembly and Differential Expression* with Homo Sapiens/hg19 (RefSeq) as reference genome. The sequence alignments and coverage were visualized in Integrative Genomics Viewer (IGV; Broad Institute, Cambridge, MA, USA).^57, 58^

### Statistical analysis

We used two-tailed *t*-test for data analysis in comparison between two groups and as *post hoc* analysis after analysis of variance in comparison among multiple groups. All experiments were repeated at least twice with at least three biological samples. We applied GraphPad Prism 6 software (GraphPad Software, Inc., La Jolla, CA, USA) for data analysis and figure preparations. A threshold of *P*<0.05 was defined as statistically significant. To identify the most relevant pathways using the RNASeq data, we selected differentially expressed genes using the following thresholds: log of fold change >0.6 and *P*-value<0.05, and applied these selected gene set for further analysis using a pathway impact analysis.^59^

## Figures and Tables

**Table 1:** Top 10 of the most impacted biological pathways

*Biological pathways*	P*-value*
Cytokine–cytokine receptor interaction	1.120e–8
CAMs	1.464e–7
Hematopoietic cell lineage	1.516e–7
Rheumatoid arthritis	3.477e–7
Axon guidance	4.534e–7
Staphylococcus aureus infection	1.038e–5
Complement and coagulation cascades	1.381e–5
ECM–receptor interaction	1.807e–5
PI3K-Akt signaling pathway	1.928e–5
Calcium signaling pathway	3.396e–5

Abbreviations: CAM, cell adhesion molecule; ECM, extracellular matrix; KO, knockout; PI3K, phosphatidylinositol 3 kinase; WT, wild type.

Pathway impact analysis for genes differentially expressed (*P*-value<0.05) in WT and KO1 cell lines identified ‘CAMs’ and ‘ECM–receptor interaction’ as 2 of the top 10 impacted biological pathways. The raw RNA sequences data have been uploaded to Figshare.
