# Supplementary material for: Metformin inhibits SUV39H1-mediated migration of prostate cancer cells
Source: Oncogenesis. 2017 May 1;6(5):e324–. doi: 10.1038/oncsis.2017.28 (PMC5523061; doi:10.1038/oncsis.2017.28)
Supplement: Supplementary Table 1 [file oncsis201728x1.docx]

**Table S1.** The transcription factors (TFs) serve as suppressors.

| Transcription factors | Fold change | | p-value | Ref. |
| --- | --- | --- | --- | --- |
| MXI1 | 1.9 | 2.88E-04 | | (1, 2) |
| MAZ | 1.8 | 2.88E-04 | | (3) |
| E2F6 | 1.6 | 5.37E-04 | | (4, 5) |
| ZNF263 | 1.5 | 7.76E-04 | | (6) |
| MYC | 1.4 | 1.22E-03 | | (7-11) |
| REST | 1.3 | 4.28E-02 | | (12, 13) |

Note: Above transcription factors were previously reported as suppressors of gene transcription and were up-regulated significantly in PC-3 SUV39H1-KO cells to WT cells according to RNA-seq data. Meanwhile, all of them may bind to the proximal promoter regions of ITGAV and ITGB1 according to Transcription Factor ChIP-seq from ENCODE with Factorbook Motifs (14-16).

1. Lee TC, Ziff EB. Mxi1 is a repressor of the c-Myc promoter and reverses activation by USF. The Journal of biological chemistry. 1999;274(2):595-606.

2. Manni I, Tunici P, Cirenei N, Albarosa R, Colombo BM, Roz L, et al. Mxi1 inhibits the proliferation of U87 glioma cells through down-regulation of cyclin B1 gene expression. Br J Cancer. 2002;86(3):477-84.

3. Izzo MW, Strachan GD, Stubbs MC, Hall DJ. Transcriptional repression from the c-myc P2 promoter by the zinc finger protein ZF87/MAZ. The Journal of biological chemistry1999. p. 19498-506.

4. Trimarchi JM, Fairchild B, Verona R, Moberg K, Andon N, Lees JA. E2F-6, a member of the E2F family that can behave as a transcriptional repressor. Proceedings of the National Academy of Sciences of the United States of America. 1998;95(6):2850-5.

5. Cartwright P, Muller H, Wagener C, Holm K, Helin K. E2F-6: a novel member of the E2F family is an inhibitor of E2F-dependent transcription. Oncogene. 1998;17(5):611-23.

6. Frietze S, Lan X, Jin VX, Farnham PJ. Genomic targets of the KRAB and SCAN domain-containing zinc finger protein 263. The Journal of biological chemistry. 2010;285(2):1393-403.

7. Roy AL, Carruthers C, Gutjahr T, Roeder RG. Direct role for Myc in transcription initiation mediated by interactions with TFII-I. Nature. 1993;365(6444):359-61.

8. Li LH, Nerlov C, Prendergast G, MacGregor D, Ziff EB. c-Myc represses transcription in vivo by a novel mechanism dependent on the initiator element and Myc box II. The EMBO journal. 1994;13(17):4070-9.

9. Philipp A, Schneider A, Vasrik I, Finke K, Xiong Y, Beach D, et al. Repression of cyclin D1: a novel function of MYC. Molecular and cellular biology. 1994;14(6):4032-43.

10. Lee LA, Dolde C, Barrett J, Wu CS, Dang CV. A link between c-Myc-mediated transcriptional repression and neoplastic transformation. The Journal of clinical investigation. 1996;97(7):1687-95.

11. Marhin WW, Chen S, Facchini LM, Fornace AJ, Jr., Penn LZ. Myc represses the growth arrest gene gadd45. Oncogene. 1997;14(23):2825-34.

12. Schoenherr CJ, Anderson DJ. The neuron-restrictive silencer factor (NRSF): a coordinate repressor of multiple neuron-specific genes. Science. 1995;267(5202):1360-3.

13. Chong JA, Tapia-Ramirez J, Kim S, Toledo-Aral JJ, Zheng Y, Boutros MC, et al. REST: a mammalian silencer protein that restricts sodium channel gene expression to neurons. Cell. 1995;80(6):949-57.

14. Gerstein MB, Kundaje A, Hariharan M, Landt SG, Yan KK, Cheng C, et al. Architecture of the human regulatory network derived from ENCODE data. Nature. 2012;489(7414):91-100.

15. Wang J, Zhuang J, Iyer S, Lin X, Whitfield TW, Greven MC, et al. Sequence features and chromatin structure around the genomic regions bound by 119 human transcription factors. Genome Res. 2012;22(9):1798-812.

16. Wang J, Zhuang J, Iyer S, Lin XY, Greven MC, Kim BH, et al. Factorbook.org: a Wiki-based database for transcription factor-binding data generated by the ENCODE consortium. Nucleic acids research. 2013;41(Database issue):D171-6.
