# Supplementary material for: Metformin inhibits SUV39H1-mediated migration of prostate cancer cells
Source: Oncogenesis. 2017 May 1;6(5):e324–. doi: 10.1038/oncsis.2017.28 (PMC5523061; doi:10.1038/oncsis.2017.28)
Supplement: Supplementary Figure 1 [file oncsis201728x2.pdf]

Figure S1

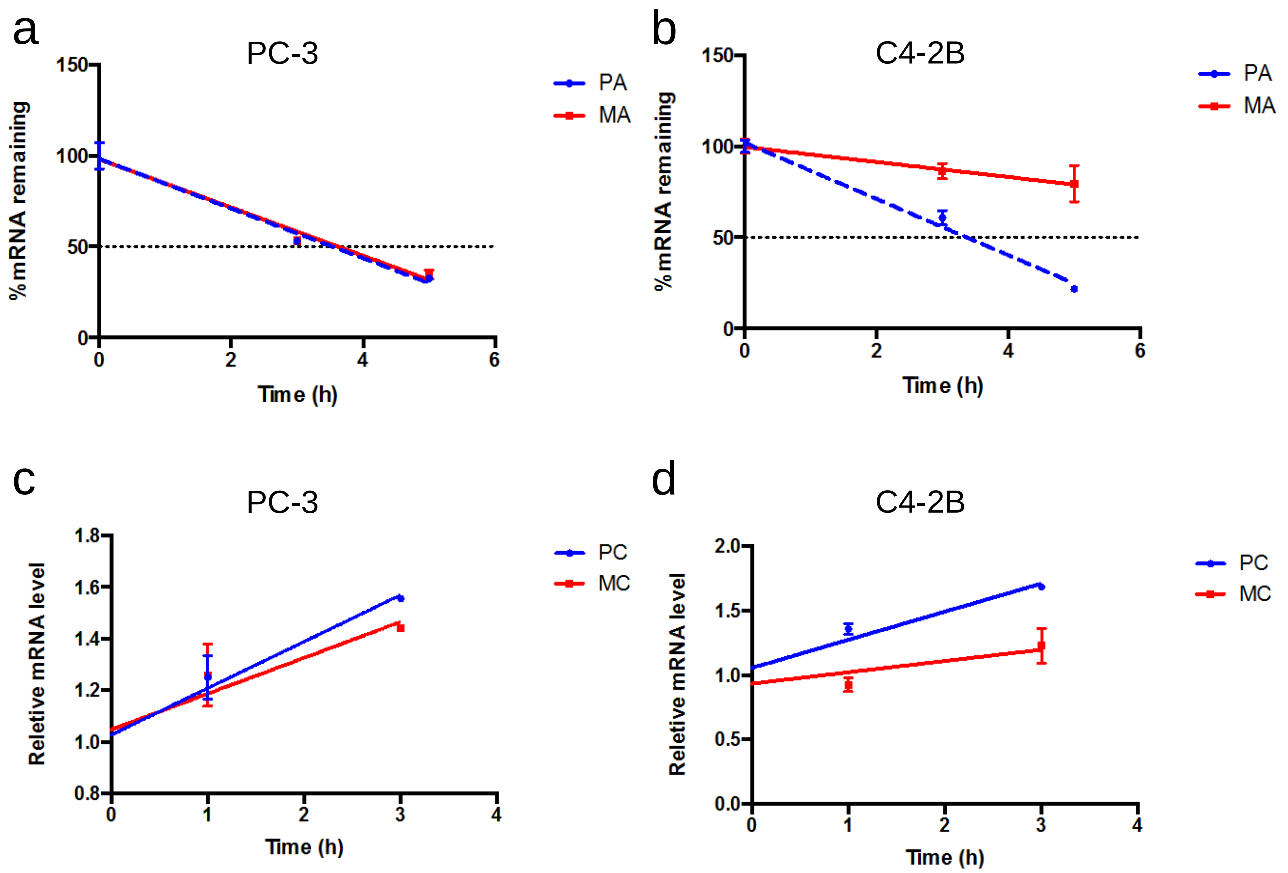

a
PC-3
150
100
50
0
%mRNA remaining
0
2
4
6
Time (h)
PA
MA
b
C4-2B
150
100
50
0
%mRNA remaining
0
2
4
6
Time (h)
PA
MA
c
PC-3
1.8
1.6
1.4
1.2
1.0
0.8
Reletive mRNA level
0
1
2
3
4
Time (h)
PC
MC
d
C4-2B
2.0
1.5
1.0
0.5
0.0
Reletive mRNA level
0
1
2
3
4
Time (h)
PC
MC

Figure S1. Metformin represses SUV39H1 mRNA transcription indirectly, but does not induce SUV39H1 mRNA stability. PCa cells, PC-3 (a) and C4-2B (b) were pre-treated with PBS or metformin (5 mM) for 24 h. Then co-treated the cells with PBS and actinomycin D (10 μg/ml) (PA), or metformin and actinomycin D (10 μg/ml) (MA). Total RNA was extracted at 0, 3, 5 h time points and the SUV39H1 mRNA levels were measured by real-time qPCR. The percentage of the remaining mRNA was normalized to the initial SUV39H1 mRNA levels at 0 h in each treatment group accordingly. The differences between the slopes of PA and MA representing the RNA degradation speed with linear regression. Metformin did not reduce SUV39H1 mRNA stability in PC-3 cells (p=0.807), while it increased the SUV39H1 mRNA stability in C4-2B (p=0.0005). PCa cells were treated with PBS (PC) or metformin (5 mM) (MC) for 48 h (PC-3) (c) and 24 h (C4-2B) (d). After medium removal and wash, fresh medium with Cycloheximide (CHX) (50 μg/ml) were added. SUV39H1 mRNA levels was measured by real-time qPCR at 0, 1, 3 h time points and normalized to its level at 0 h. Using linear regression, we determined the differences between the slopes of PC and MC as the measure of transcription rates after the removal of metformin the inhibition of de novo protein synthesis affects the restoration of SUV39H1 transcription. In PC-3, the difference between the slopes was not significant (P=0.3526) while in C4-2B was significant (P=0.0354). This result indicates that the reduction of SUV39H1 mRNA levels by metformin is likely to be indirect and relies on de novo protein synthesis at least in C4-2b cells.
