# Supplementary material for: Metformin inhibits SUV39H1-mediated migration of prostate cancer cells
Source: Oncogenesis. 2017 May 1;6(5):e324–. doi: 10.1038/oncsis.2017.28 (PMC5523061; doi:10.1038/oncsis.2017.28)
Supplement: Supplementary Figure 2 [file oncsis201728x3.pdf]

Figure S2

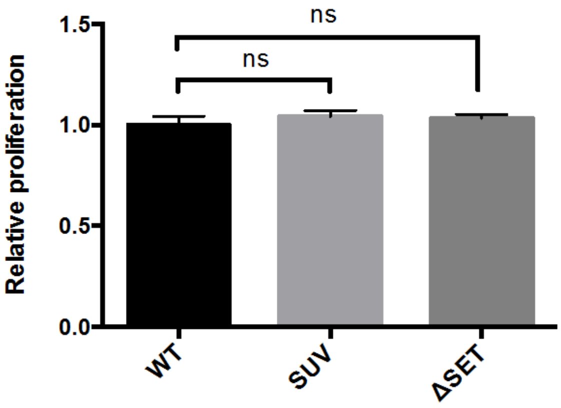

Figure S2. Cell proliferation of C4-2B WT, SUV39H1 and SUV39H1ΔSET over-expressing cells. Cells were plated in triplicate in 48-well plates (2×10^4 cells/well). After 72 h, cells were stained with crystal violet followed by OD measurement. Relative proliferation was calculated with the OD values normalized to control WT cells. Data shown are mean ± SEM ($n\geq3$). ns, no significant.
