# Supplementary material for: Metformin inhibits SUV39H1-mediated migration of prostate cancer cells
Source: Oncogenesis. 2017 May 1;6(5):e324–. doi: 10.1038/oncsis.2017.28 (PMC5523061; doi:10.1038/oncsis.2017.28)
Supplement: Supplementary Figure 3 [file oncsis201728x4.pdf]

Figure S3

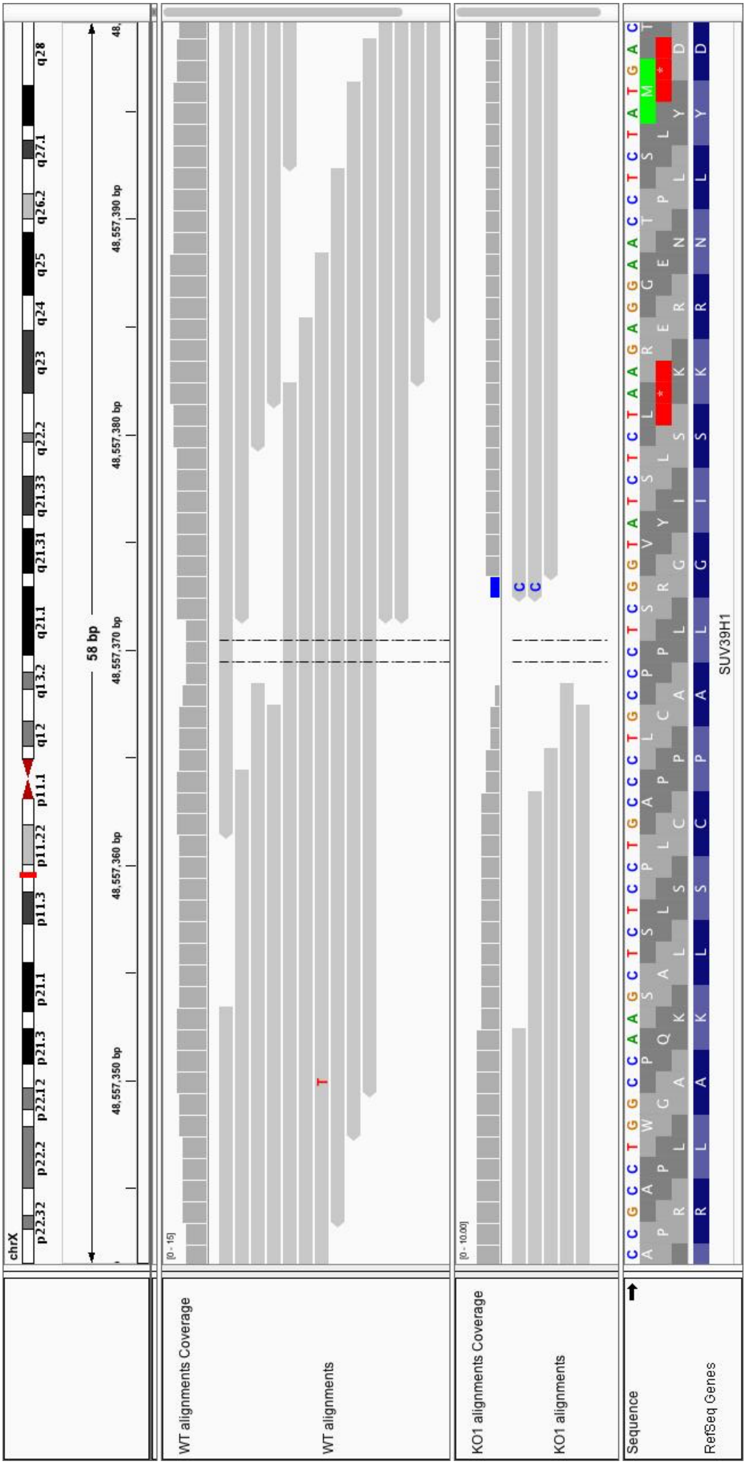
chrX
58 bp
48,557,350 bp
48,557,360 bp
48,557,370 bp
48,557,380 bp
48,557,390 bp
WT alignments Coverage
WT alignments
KO1 alignments Coverage
KO1 alignments
Sequence
RefSeq Genes
SUV39H1

Figure S3. Deletions in exon 2 of SUV39H1 generated by CRISPR-Cas9. RNA-seq of PC-3 WT and KO1 cells shows the reads alignments and coverage in exon 2 targeting region of SUV39H1, showing the expected deletions near the PAM motif in the KO cells.
