# Supplementary material for: Metformin inhibits SUV39H1-mediated migration of prostate cancer cells
Source: Oncogenesis. 2017 May 1;6(5):e324–. doi: 10.1038/oncsis.2017.28 (PMC5523061; doi:10.1038/oncsis.2017.28)
Supplement: Supplementary Figure 4 [file oncsis201728x5.pdf]

Figure S4

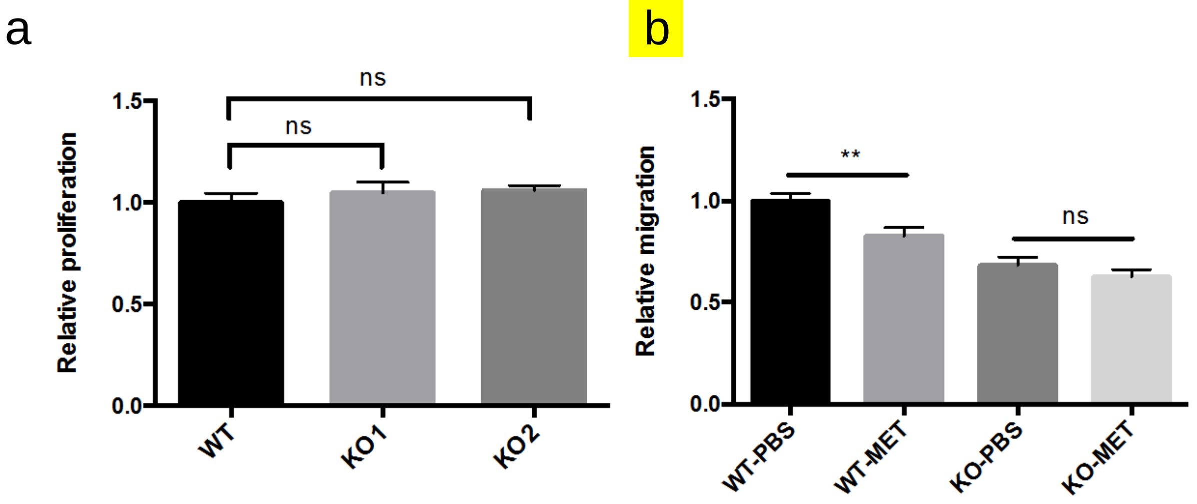

Figure S4. (a). Cell proliferation of PC-3 WT and SUV39H1-KO cell lines (KO1 and KO2) cells. PC-3 WT, KO1 and KO2 cells were plated in triplicate in 48-well plates (1.5×10^4 cells/well). Cell proliferation was measured via crystal violet staining followed by OD value reading. Relative cell proliferation was calculated with the OD values normalized to WT cells. Data shown are mean ± SEM (*n*=3). ns, no significant. (**b**). Cell migration was measured with wound healing assay in WT and KO cells with PBS or metformin treatments. Data shown are mean ± SEM (*n*=3). ** $p<0.01$, ns, no significant.
