# Supplementary material for: Metformin inhibits SUV39H1-mediated migration of prostate cancer cells
Source: Oncogenesis. 2017 May 1;6(5):e324–. doi: 10.1038/oncsis.2017.28 (PMC5523061; doi:10.1038/oncsis.2017.28)
Supplement: Supplementary Figure 5 [file oncsis201728x6.pdf]

Figure S5

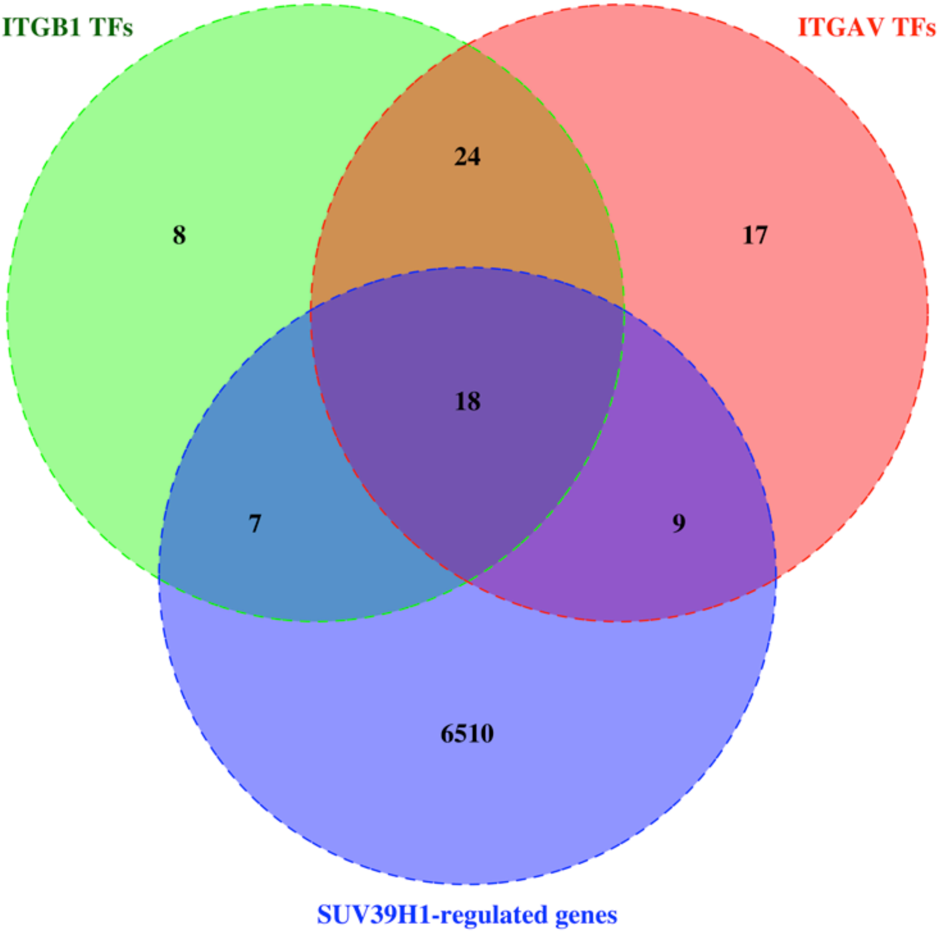

Figure S5. Venn Diagram of SUV39H1-regulated transcription factors that can bind to the promoter regions of ITGAV and ITGB1. Eighteen transcription factors were found from 6544 SUV39H1-regulated genes and transcription factors binding to promoters of ITGAV (ITGAV TFs) and ITGB1 (ITGB1 TFs) accordingly.
